# Study of the influence of population immunity to tick-borne encephalitis virus on the characteristics of the epidemic process in Russia

**DOI:** 10.3389/fimmu.2025.1525388

**Published:** 2025-08-08

**Authors:** Temur K. Muminov, Vladimir A. Gushchin, Denis A. Kleymenov, Artem P. Tkachuk, Viktor A. Manuylov, Andrei E. Siniavin, Daria A. Ogarkova, Nadezhda A. Kuznetsova, Vladimir I. Zlobin, Alexander L. Gintsburg

**Affiliations:** ^1^ Federal State Budget Institution “National Research Centre for Epidemiology and Microbiology Named after Honorary Academician N. F. Gamaleya” of the Ministry of Health of the Russian Federation, Moscow, Russia; ^2^ Department of Medical Genetics, I. M. Sechenov First Moscow State Medical University, Moscow, Russia; ^3^ Department of Virology, Lomonosov Moscow State University, Moscow, Russia; ^4^ Federal State Budgetary Institution, National Medical Research Center for Phthisiopulmonology and Infectious Diseases, Ministry of Health of the Russian Federation, Moscow, Russia; ^5^ Department of Natural Sciences, Novosibirsk State University, Novosibirsk, Russia; ^6^ Shemyakin-Ovchinnikov Institute of Bioorganic Chemistry of the Russian Academy of Sciences, Moscow, Russia; ^7^ Infectiology Department, I. M. Sechenov First Moscow State Medical University, Moscow, Russia

**Keywords:** anti-TBEV antibodies, seroepidemiology, prevalence, immunological surveillance, serological monitoring, vaccines, Russian Federation

## Abstract

**Background:**

Tick-borne encephalitis (TBE) is a significant public health challenge in Russia. Vaccination is one of the most effective measures to control TBE. The aim of our study was to assess the state of anti-TBE virus population immunity, including artificial post-vaccine and natural post-infection immunity, in the context of characteristics of the epidemic process in Russia.

**Materials and methods:**

During the period from 2018 to 2020, we studied 28,395 conditionally healthy volunteers from various regions of Russia, without age and gender restrictions. Blood serum samples were tested for anti-TBE virus IgG antibodies. All volunteers completed questionnaires to collect demographic data, information about residence, TBE vaccination, and tick bites.

**Results:**

Our study included participants from non-endemic (Moscow and the Republic of Dagestan) and endemic regions (Moscow Region, St. Petersburg, Leningrad Region, Novosibirsk Region, Khabarovsk Region). In regions with the highest protection against TBE, such as Novosibirsk Region and Khabarovsk Region, the proportions of individuals with seropositive and protective antibody titers were below 45% and 35%, respectively. The lowest rate of protective immunity was found among children (25.4% in Novosibirsk Region and 22% in Khabarovsk Region) and those aged 60 and older (27.3% and 25.1%, respectively). Situation was even more challenging in St. Petersburg and Leningrad Region, where the rate of protective antibody titers ranged from 4.3% to 8.7%. The highest vaccination coverage was found among volunteers from Novosibirsk Region and Khabarovsk Region: 32.5% and 27.4%. In St. Petersburg and Leningrad Region, vaccination coverage was ranged from 10.4% to 11.3%, while in other regions it was below 6%. The rates of post-vaccine protective immunity were 63.1% in Khabarovsk Region, 71.6% in Novosibirsk Region and up to 50% in other regions. The rates of post-infection immunity were 33.4% in Novosibirsk Region, 42.4% in Khabarovsk Region and below 12% in other regions.

**Conclusions:**

Our results demonstrated diversity of population immunity level and structure in different regions of Russia. The analysis showed that study participants are at risk of TBE infection, especially high in endemic regions, due to insufficient level of population immunity, vaccination coverage, and protective post-vaccine immunity.

## Introduction

Tick-borne encephalitis (TBE) is one of the most dangerous naturally focal transmissible infections, making it a significant public health concern in Russia. The current burden of TBE is characterized by a wide area of pathogen circulation with no clear evidence of a decrease of TBE incidence and mortality rates in the Russian Federation (1, 2). Despite the decreasing trend since the early 21st century, a significant increase in the incidence rates was reported in 2022–2023 compared to 2020-2021 (1–3).

Due to the lack of highly effective etiological treatments, mass vaccination remains the most significant measure to reduce the burden of TBE in endemic regions, of which there are 49 in the Russian Federation according to the data from 2023 (1, 2). Several vaccines of domestic and foreign production are approved for use in Russia. Russian vaccines use strains of the Far Eastern TBEV genotype, while foreign vaccines use strains of the European TBEV subtype (1, 4).

The genetic diversity of tick-borne encephalitis virus (TBEV) strains circulating in the Russian Federation includes at least four known virus genotypes (subtypes): Far Eastern, European, Siberian, and Baikal (4). In some regions of Russia, the previously predominant Far Eastern subtype of the virus is being replaced by the Siberian subtype (5–9).

The scale of TBE vaccination coverage in Russia has more than doubled since 2002 (1, 3). However, even the current levels of immunization campaigns are insufficient to meet the requirements of Sanitary Rules and Regulations 3.3686-21 (as amended on May 25, 2022). This regulation mandates TBE vaccination for individuals up to 18 years of age and adults living in TBE-endemic administrative territories, with at least 95% coverage (10). Beyond increasing vaccination coverage, the question of improving the immunization program remains open, given the reported cases of TBE among vaccinated individuals (4).

The current dynamics of the TBE epidemic demand an in-depth, multifaceted analysis to determine the most effective measures to reduce TBE incidence and mortality rates. It is important to assess the current state of population immunity and its influence on the course and characteristics of TBE epidemic process. The population immunity against TBEV consists of two components: artificial post-vaccine immunity and natural post-infection immunity, which are difficult to differentiate due to subclinical or inapparent forms of TBE (11, 12).

The effectiveness of the existing TBE prevention strategy can be evaluated based on documented vaccination coverage, the actual vaccination rate of the population, and the results of serological monitoring, which assesses the state of population immunity and identifies susceptible and protected populations. Serological monitoring can also supplement existing morbidity data by revealing the prevalence of subclinical or inapparent TBE cases through the presence of TBEV antibodies (12).

Seroepidemiological studies on TBE over the past two decades have primarily focused on analyzing epidemic process manifestations compared to serological monitoring results in specific TBE-endemic regions of the Russian Federation. Some studies assessing the effectiveness of vaccination approaches in these regions included only controlled cohorts of vaccinated participants (13, 14). Other studies analyzed TBEV seroprevalence based on data from non-vaccinated populations (15–17). A number of studies have examined population immunity in specific TBE-endemic regions such as the Urals, Siberia, Transbaikal, and Southern Russia, providing data on the level, intensity, and structure of population immunity (18–22). Most of these studies included only adult participants, primarily blood donors from the respective regions. A significant limitation of these studies was the absence of data on tick bites, a key TBE risk factor, making it impossible to distinguish between the post-vaccine and post-infection components of population immunity. Many previous serological monitoring studies were limited by their analysis of seroprevalence using a threshold antibody titer of 1:100, which is now considered a marker of immunological memory. However, the lower protective antibody level threshold is a titer of 1:400 (14, 23–25).

The objective of our study was to assess the state and structure of population immunity against TBEV, including distinction between artificial post-vaccine and natural post-infection immunity, and to analyze the influence of population immunity on the characteristics of the epidemic process.

## Materials and methods

### Study population and serum sampling design

In this study, we established a biobank with 28,395 blood serum samples collected from conditionally healthy individuals in the Russian Federation without age restrictions from 2018 to early 2020. The whole study was conducted in accordance with the principles expressed in the World Medical Association Declaration of Helsinki regarding ethical medical research involving human subjects. Written informed consent was obtained from all participants. The study design was approved by the Independent Interdisciplinary Ethics Committee for the Ethical Review of Clinical Research, Moscow, Russia (Approval No. 17 dated November 16, 2019).

The biobank was established by a working group at the Federal State Budget Institution National Research Centre for Epidemiology and Microbiology named after Honorary Academician N.F. Gamaleya of the Ministry of Health of the Russian Federation. Enrollment was carried out in five geographical regions of Russia, selected to maximize the diversity of climatic, geographical, and socio-economic conditions. The regions included were Moscow Region (including the capital city, Moscow), the city of St. Petersburg and Leningrad Region (combined into St. Petersburg Region), Novosibirsk Region, the Republic of Dagestan, and Khabarovsk Region.

The collection of samples for the study was performed using disposable vacuum blood collection systems, which have a registration certificate and are authorized for medical use in the Russian Federation. Sample preparation, transportation, and temporary storage of samples were performed under conditions that ensured the preservation of their biological properties.

The volume of whole blood collected from volunteers was 2.5 milliliters (ml) for participants under one year of age, 3.5 ml for those aged 1–7 years, 7 ml for those aged 8–12 years, and 10.5 ml for those aged 13 years and older.

For long-term biobank storage, collected serum samples were labeled, aliquoted into 500 µL barcode-labeled tubes, and archived in a low-temperature biobank at -80°C with automatic sample operation available at the National Research Centre for Epidemiology and Microbiology named after Honorary Academician N.F. Gamaleya.

### Serologic testing

We tested collected blood serum samples for the presence of TBEV IgG antibodies by the enzyme-linked immunosorbent assay (ELISA) using commercially available serological kits D-1156 VectoTBE-IgG (Vector-Best, Russia) with a maximum antibody titer of 1:1600. Serologic testing of serum samples was performed as biological material was collected at the laboratory of the National Research Centre for Epidemiology and Microbiology named after Honorary Academician N.F. Gamaleya. The serologic testing was completed in March 2020, and the tests were performed according to the manufacturer’s instructions, which set the threshold IgG antibody titer to TBEV as ≥1:100. However, when analyzing the obtained seroepidemiological data, we also used a titer of 1:400 as a threshold value, as it is considered to be the lower limit of immunological protection (14, 23–25). Some participants indicated a permanent residence outside the selected regions, so their data were excluded from further analysis.

### Questionnaire

All study participants completed questionnaires containing blocks of questions on demographic data, place of birth and residence, health status (including vaccination history and diseases), behaviors and risk factors for viral infections (including tick bites), as well as education and marital status. The vaccination section did not include information on the type of vaccine used, the time of the last vaccination, immunization schedule violations, and booster vaccinations. Information on previous TBE infections was not collected in the section on past illnesses.

### Data analysis and statistical processing

To study population immunity characteristics and structure in various cohorts, we analyzed questionnaire data on age, region of residence, previous TBE vaccination, and history of tick bites. Given the naturally focal nature of TBE, the primary stratification in our analysis was by region of residence.

Information on previous immunization against TBE among seropositive individuals was used to assess the structure of population immunity. The contribution of post-vaccine antibodies to the formation of the immune layer was assessed by the proportion of vaccinated among seropositive study participants. The role of natural TBE infection in the structure of population immunity was assessed by the proportion of seropositive individuals without vaccination. To evaluate post-vaccine immunity, we analyzed serum seropositivity only from vaccinated volunteers who reported no history of tick bites to minimize the risk of bias due to post-infection antibodies.

Due to the lack of information regarding TBE history and the high prevalence of febrile, subclinical, and inapparent infection forms, we investigated natural post-infection immunity by assessing the seropositivity of participants who reported tick bites and no TBE vaccination.

We analyzed seroepidemiological indicators in the child age group for the entire population of persons under 18 years of age, as some age groups in certain regions did not reach the planned cohort size (26).

The comparison of seroepidemiological trends in our results with TBE epidemic manifestations was made using official data on incidence and virus carriage from accessible sources, such as the official websites of territorial sanitary and epidemiological supervision departments and publications in reviewed scientific and medical journals containing state statistical data on infectious and parasitic diseases.

We used the following statistical methods to describe the study data: seroprevalence was calculated as the proportion of positive samples on anti-TBE antibodies, 95% confidence intervals (95% CI), and Pearson’s chi-squared test for comparing independent samples. Differences were considered statistically significant at p ≤ 0.05. All calculations were performed using Microsoft Excel 2016.

## Results

### Study population and demographic data

Our study resulted in the establishment of a biobank containing blood serum samples from 28,395 conditionally healthy participants residing in selected regions, with known quantitative ELISA results for TBEV IgG antibodies. The sample distribution was as follows: Moscow Region - 7,298 samples, St. Petersburg Region - 5,195, Novosibirsk Region - 8,069, Republic of Dagestan - 4,014, and Khabarovsk Region - 3,819 samples.

**Figure 1:**
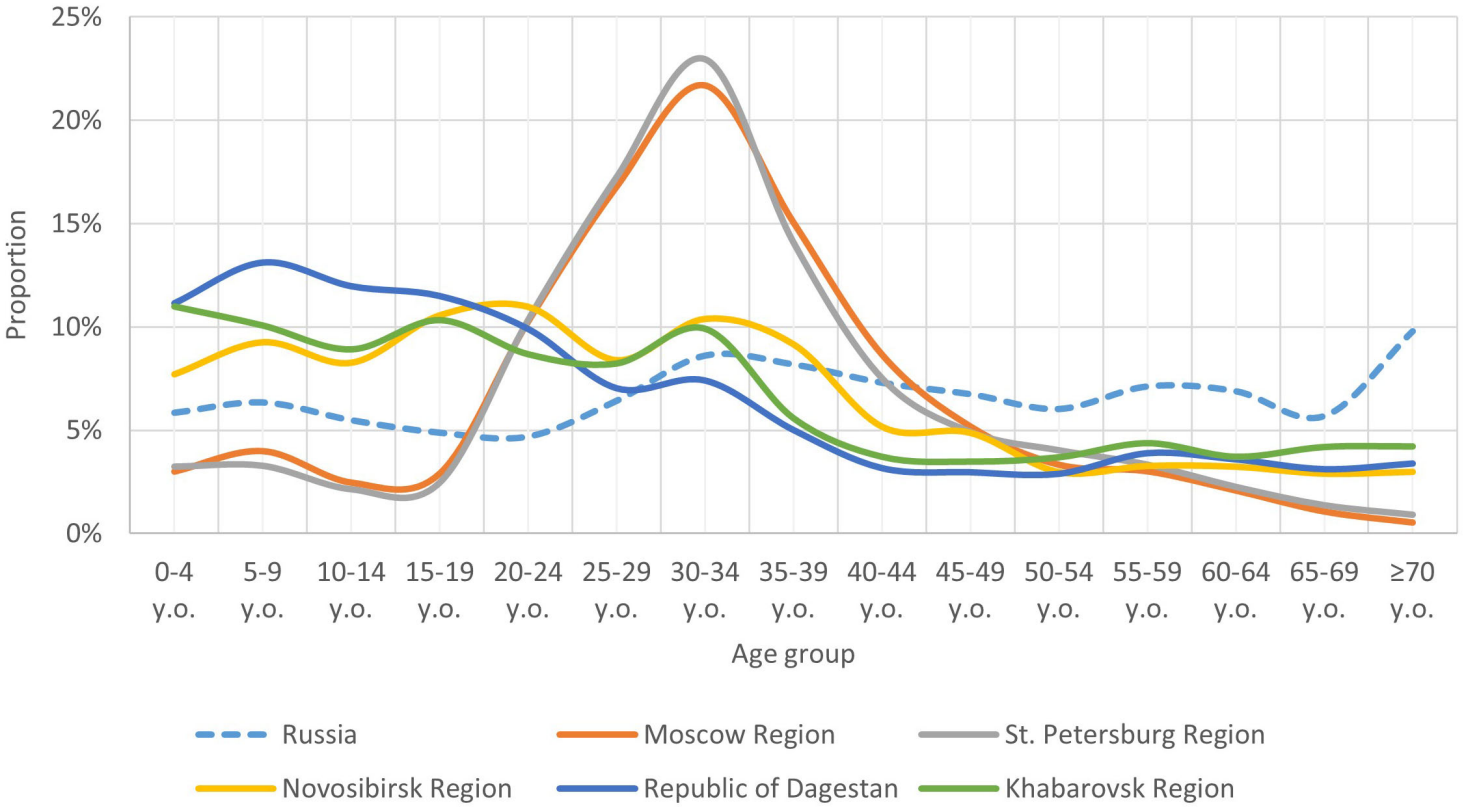
Comparison of the age structure (share of individual age groups) of the population of the Russian Federation with the age structure of the study participants in individual regions.

When comparing the age structure of volunteer samples from different regions (**Figure 1**), we found that some age groups were unevenly represented, with proportions differing between regions and not fully reflecting the age structure of the Russian population as of early 2020 (**Supplementary Table 1**) (27).

Notably, certain child age subgroups were comparably proportionate within each region, though significant differences in child population proportions were observed between regions. In the Novosibirsk Region, Republic of Dagestan, and Khabarovsk Region, the child population was comparable and higher than the overall Russian average. In Moscow Region and St. Petersburg Region, the child population proportion was comparable but lower than the Russian average.

The adult population in our study exhibited significant regional age structure heterogeneity, particularly between young adults (18–44 years), middle-aged adults (45–59 years), and older adults. Consequently, our population immunity level analysis was conducted separately for these adult age groups.

The proportion of young adults was higher in Moscow Region and St. Petersburg Region, compared to other regions and the Russian average. In the other regions, this age group’s proportion was comparable to the Russian average.

The proportion of the middle-aged group was similar across all regions and aligned with the Russian average. The older age group’s proportion was slightly lower in Moscow Region and St. Petersburg Region, but comparable in other regions and aligned with the country average.

According to official statistics, TBE-endemic territories were identified as follows: Moscow Region (2 administrative territories out of 53), the city of St. Petersburg (6 administrative territories out of 18), Leningrad Region (all administrative territories), Novosibirsk Region (23 administrative territories out of 33), and Khabarovsk Region (16 administrative territories out of 19) (28). The city of Moscow and the Republic of Dagestan are non-endemic regions (28).

Our analysis of study participants’ residence in TBE-endemic administrative territories is shown in **Table 1**. Information for volunteers whose residence endemicity could not be determined was excluded. In the Moscow Region group, only 0.8% lived in the endemic Taldom and Dmitrov districts of the Moscow Region. All participants from St. Petersburg Region were considered to reside in an endemic area due to the lack of specific district residence data within the city of St. Petersburg. All study participants from the Republic of Dagestan lived in non-endemic areas. In the Novosibirsk Region and Khabarovsk Region, 97.4% and 99.9% of volunteers, respectively, resided in endemic regions.

**Table 1:** Distribution of participants according to their place of residence among the endemic territories.

Region	Endemic territories		Non-endemic territories		Unknown		p value
	Number of participants, n	Share in the region, %	Number of participants, n	Share in the region, %	Number of participants, n	Share in the region, %	
Moscow Region	56	0.8%	7225	99.0%	17	0.2%	< 0.01
St. Petersburg Region*	5195	100.0%	0	0.0%	0	0.0%	
Novosibirsk Region	7838	97.1%	206	2.6%	25	0.3%	
Republic of Dagestan	0	0.0%	4014	100.0%	0	0.0%	
Khabarovsk Region	3809	99.7%	2	0.1%	8	0.2%	

*Since information on the areas of residence of the study participants within the city was not collected during the questionnaire, it is not possible to specify the proportion of study participants from St. Petersburg who lived in the city’s TBE endemic areas.

### Population immunity analysis

**Figure 2:**
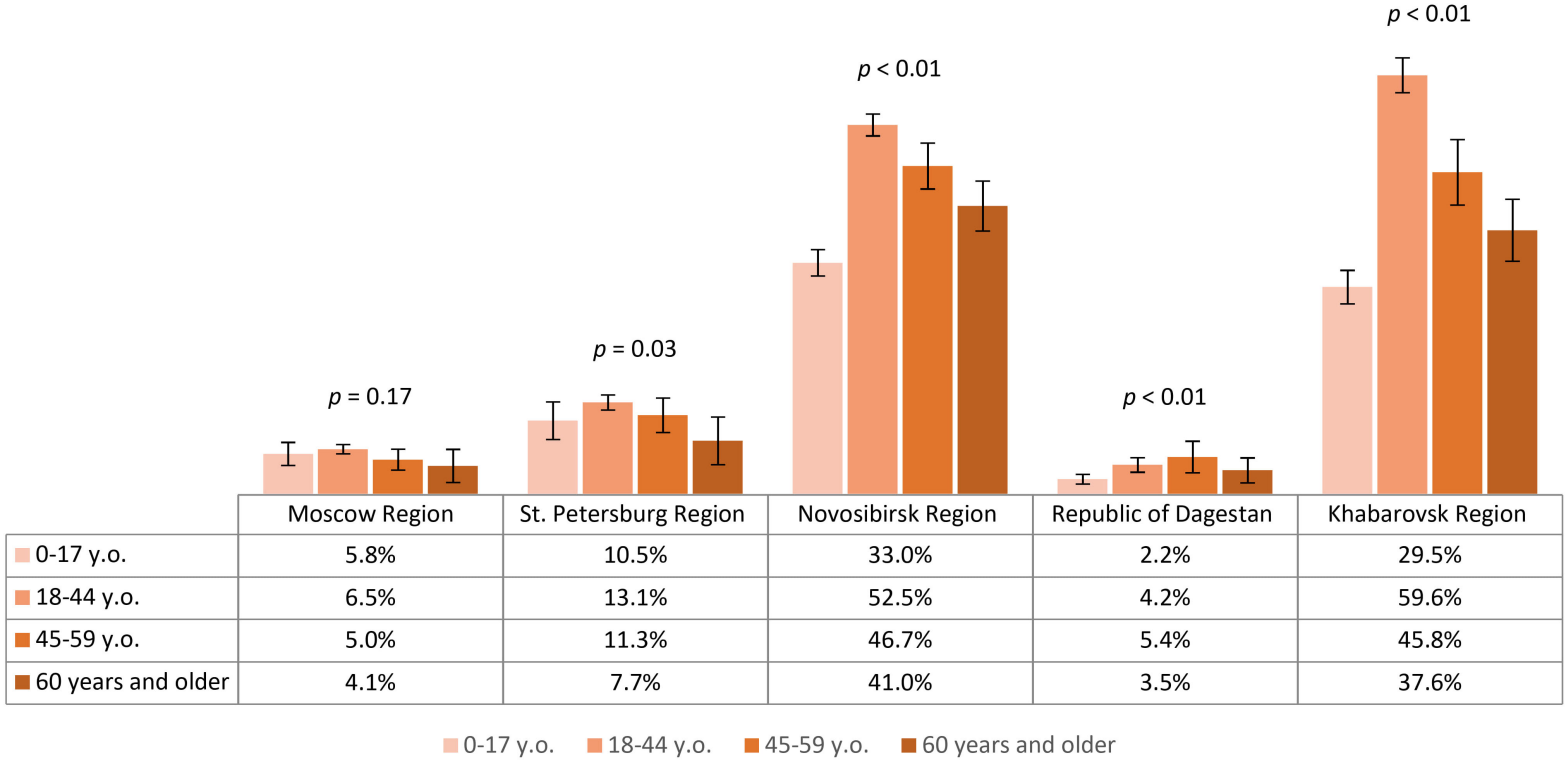
Proportion of seropositive study participants in different age groups by region.

The prevalence of TBEV IgG antibodies (titer 1:100 or higher) in our cohort was 23.0% (95% CI: 22.6-23.5), including 6.1% (95% CI: 5.6-6.7) in Moscow Region, 12.4% (95% CI: 11.5-13.3) in St. Petersburg Region, 44.9% (95% CI: 43.8-46.0) in Novosibirsk Region, 3.4% (95% CI: 2.8-3.9) in the Republic of Dagestan, and 44.3% (95% CI: 42.7-45.9) in Khabarovsk Region. Regional differences were statistically significant except between Novosibirsk Region and Khabarovsk Region (p=0.54). The seropositive proportion (titer 1:100 or higher) in different age groups by region is shown in **Figure 2**. In all regions except Moscow Region, TBEV antibody prevalence significantly differed among age groups.

To assess the contribution of vaccination and acquired infection to population immunity, we analyzed the proportion of vaccinated individuals among the seropositive population of study participants. The results are shown in **Table 2**. In all regions except the Republic of Dagestan, vaccinated volunteers constituted a substantial portion of the seropositive individuals. Analysis showed a statistically significant difference in the proportion of vaccinated seropositive individuals in Khabarovsk Region (47.8%) compared to Moscow Region (55.6%), Novosibirsk Region (60.9%), and St. Petersburg Region (61.2%).

**Table 2:** Proportion of vaccinated and unvaccinated study participants among those with anti-TBEV antibodies.

Population	Vaccinated, % (95% CI)	Non-vaccinated, % (95% CI)	p value
Moscow Region	55.6% (51.0-60.2)	44.4% (39.8-49.0)	< 0.01
St. Petersburg Region	61.2% (57.4-65.0)	38.8% (35.0-42.6)	
Novosibirsk Region	60.9% (59.3-62.5)	39.1% (37.5-40.7)	
Republic of Dagestan	5.1% (1.4-8.8)	94.9% (91.2-98.6)	
Khabarovsk Region	47.8% (45.4-50.2)	52.2% (49.8-54.6)	

**Figure 3:**
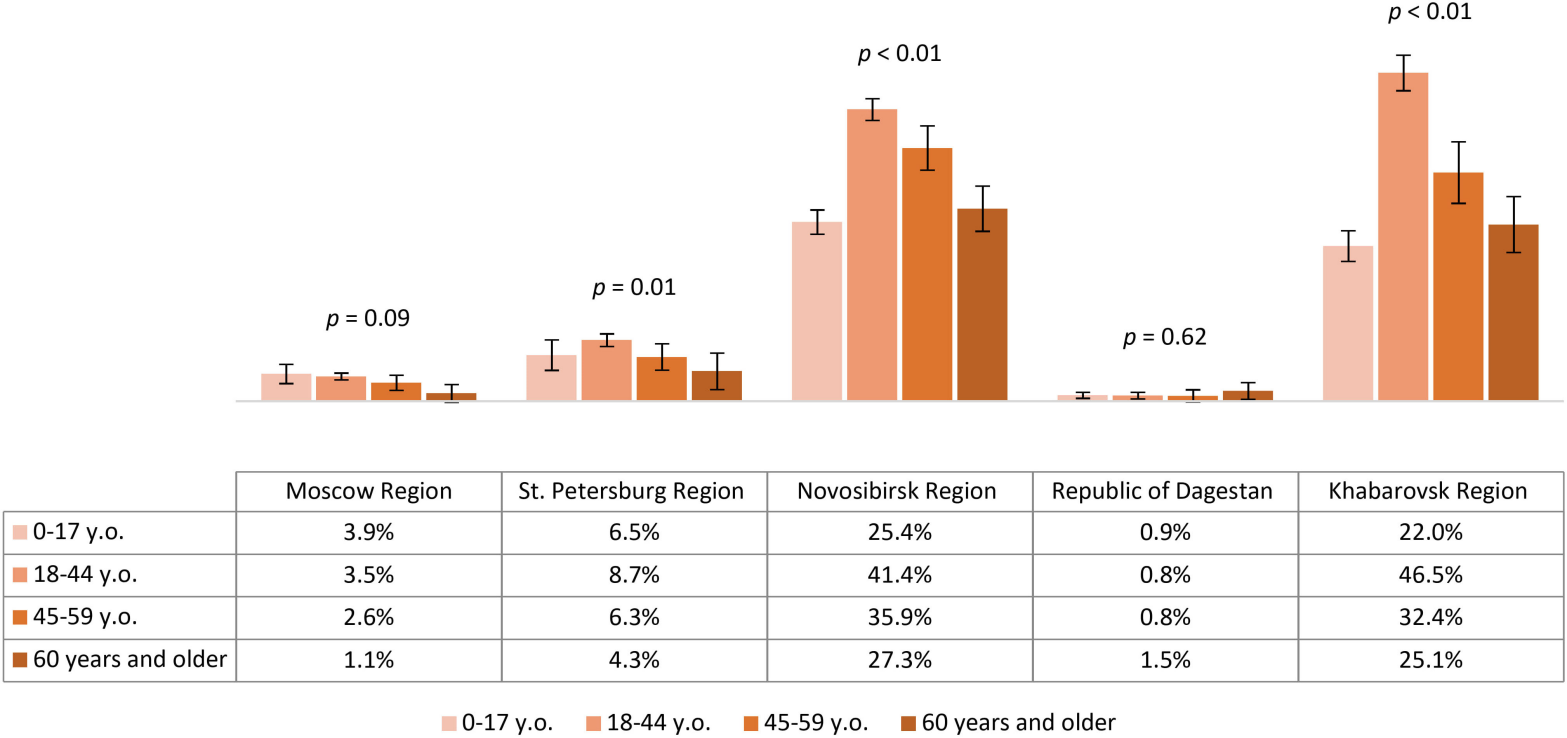
Proportion of study participants with protective antibody titer (1:400) in different age groups by region.

The analysis we conducted on the proportion of participants with protective antibody titers (assuming that protective titer is 1:400 and above) showed the highest level of protection among volunteers from Novosibirsk Region – 34.6% (95% CI: 33.6-35.7) and Khabarovsk Region – 33.3% (95% CI: 31.8-34.8), with comparable results (p=0.16). Results in other regions were significantly lower and varied statistically among themselves, amounting to 8.0% (95% CI: 7.2-8.7) in St. Petersburg Region, 3.4% (95% CI: 2.9-3.8) in Moscow Region and 0.9% (95% CI: 0.6-1.2) in the Republic of Dagestan. The level of population immunity in different age groups by region is shown in **Figure 3**. It is noteworthy that in the relatively more protected Novosibirsk Region and Khabarovsk Region, the analyzed values significantly differed among age groups: the highest proportion of individuals with protective antibody titers was found in the 18–44 years group, and the lowest results were observed in the child population and the 60 years and older group.

Based on this analysis, IgG antibodies to TBEV were detected among volunteers residing permanently in all studied regions, predominantly in the 18–44 and 45–59 year age groups. The contribution of vaccination to the seropositive layer was lowest in the non-endemic Republic of Dagestan. In analyzing the state of population immunity, we found a low prevalence of protective antibody titers, even in the endemic regions included in our study: Novosibirsk Region, Khabarovsk Region, and St. Petersburg Region. The level of population immunity in the most protected regions (Novosibirsk Region and Khabarovsk Region) was lower in the child population and among the 60 years and older age group compared to participants aged 18–44 and 45-59.

### Post-vaccine immunity analysis (vaccination status according to the questionnaire)

**Figure 4:**
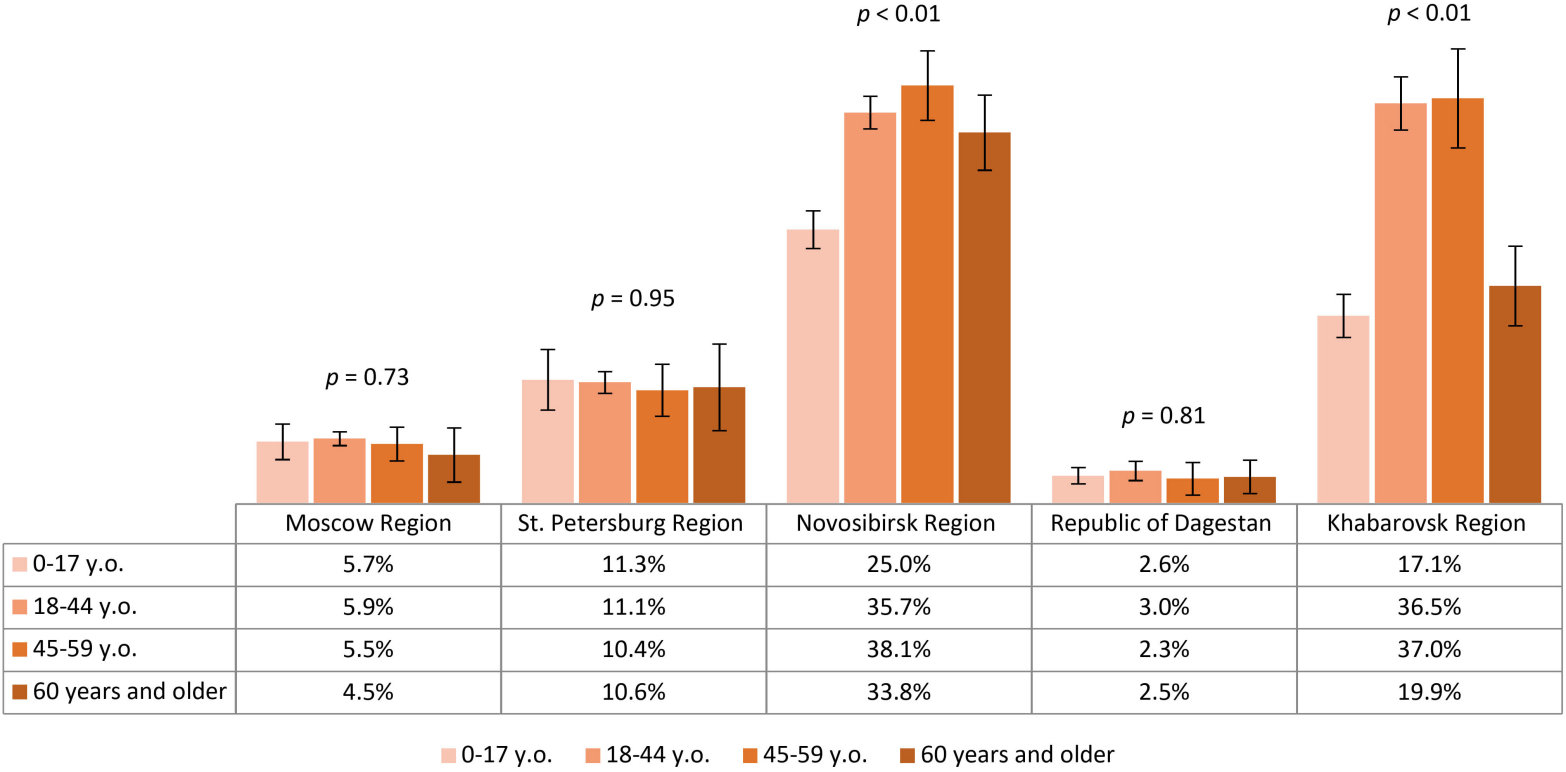
Proportion of TBE vaccinated study participants in different regions according to age groups.

The vaccination coverage of our study participants varied significantly between all regions, with rates from lowest to highest being 2.7% (95% CI: 2.2-3.2) in the Republic of Dagestan, 5.8% (95% CI: 5.3-6.3) in Moscow Region, 11% (95% CI: 10.1-11.8) in St. Petersburg Region, 27.4% (95% CI: 26.0-28.9) in Khabarovsk Region and 32.5% (95% CI: 31.5-33.6) in Novosibirsk Region. The proportion of individuals reporting TBE vaccination within different age groups across regions is shown in **Figure 4**. Our analysis showed that no age group reached 40% vaccination coverage. The relatively better situation in all age categories was seen among the study participants from Novosibirsk Region and Khabarovsk Region, with immunization coverage in children (both regions) and those aged 60 and older (in Khabarovsk Region) being significantly lower than in the 18–44 and 45–59 year age groups.

**Figure 5:**
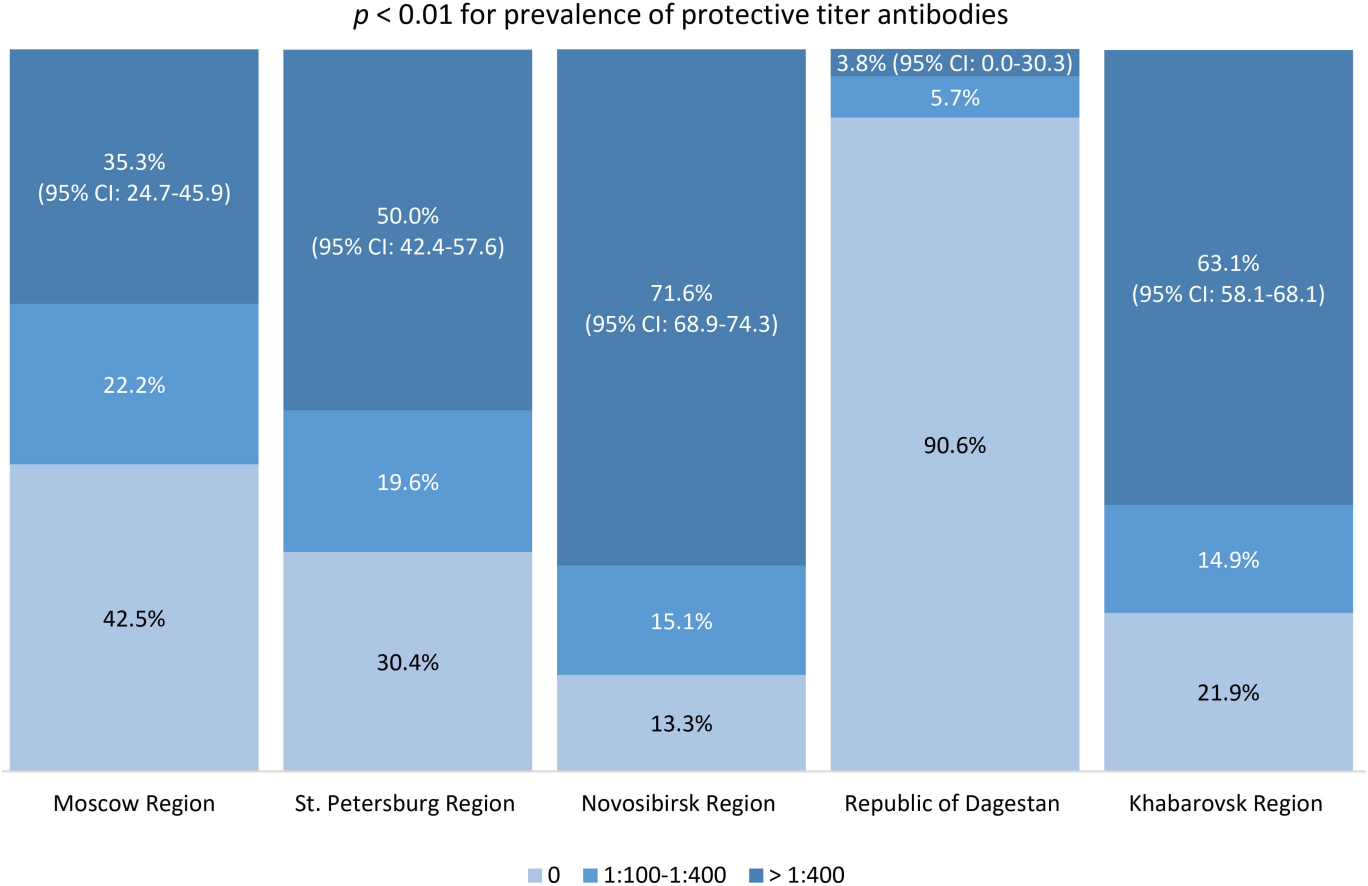
Proportion of individuals with different TBE antibody titers in the vaccinated population.

The post-vaccine immunity structure among study participants, according to IgG TBEV antibody titers by region, is shown in **Figure 5**. We identified statistically significant differences between studied regions in the proportion of participants with protective antibody titers (those with a titer of 1:400 and above). The most favorable situation in terms of protective immunity levels was found in Novosibirsk Region, Khabarovsk Region, and St. Petersburg Region: among immunized participants, the proportions of individuals with protective post-vaccine antibody titers were 71.6% (95% CI: 68.9-74.3), 63.1% (95% CI: 58.1-68.1), and 50% (95% CI: 42.4-57.6), respectively.

Our vaccination coverage analysis showed statistically significant differences between regions, with the highest results obtained in regions with a considerable share of individuals residing in endemic areas. In the children’s age groups and among those aged 60 and older in Novosibirsk Region and Khabarovsk Region, vaccination coverage was lower than in the 18–44 and 45–59 years age groups. The highest level of protective immunity (antibody titer 1:400 and above) was seen among vaccinated study participants from Novosibirsk Region and Khabarovsk Region.

### Natural post-infection immunity analysis

**Figure 6:**
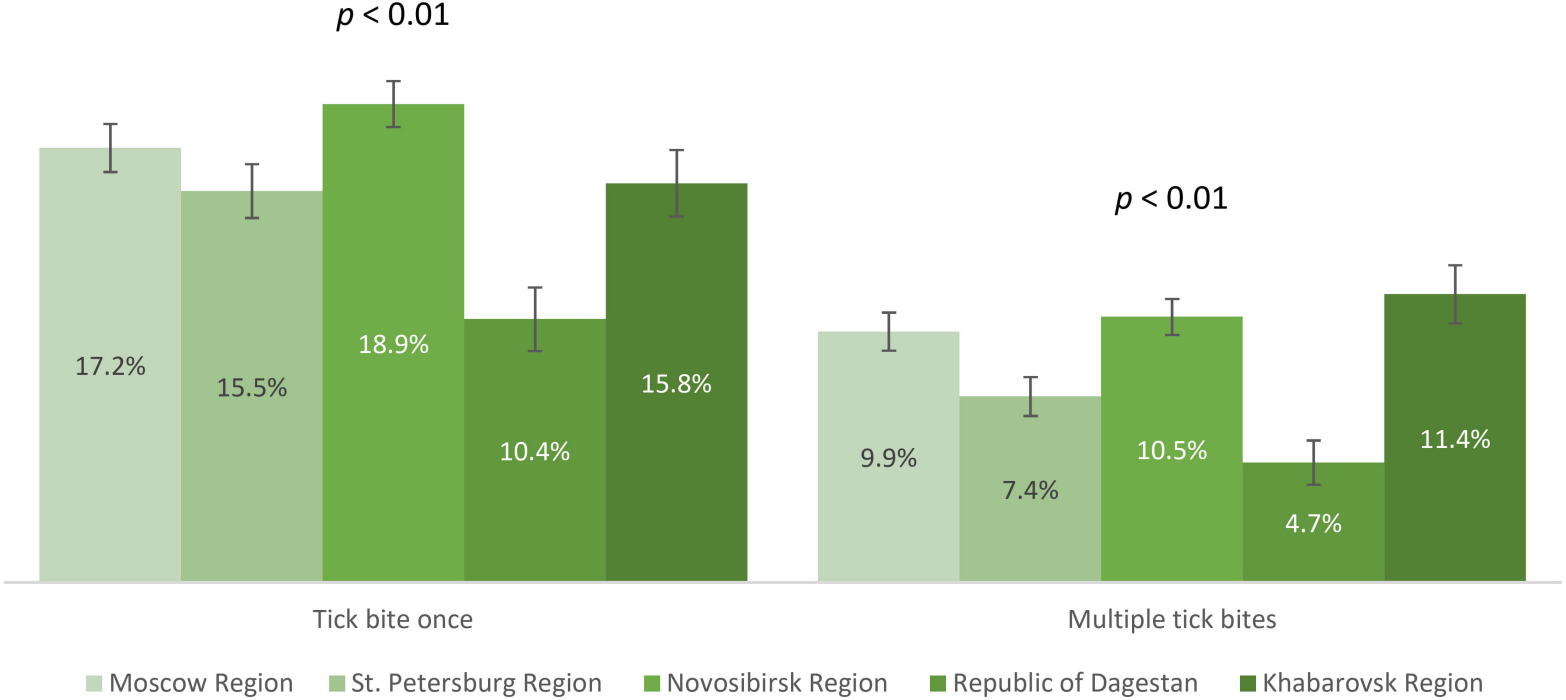
Distribution of study participants based on tick bite data.

We analyzed the contribution of natural contact with the virus among study participants who had experienced tick bites. The proportions of participants who had no tick bites, single, or multiple tick bites are shown in **Figure 6**. The proportion of study participants with a history of single and multiple tick bites differed significantly between the regions. The observed differences were primarily associated with lower rates in the Republic of Dagestan. Relatively higher proportions of individuals with tick bites (both once and multiple) were in Novosibirsk Region – 29.4% (95% CI: 28.4-30.5), Khabarovsk Region – 27.2% (95% CI: 25.6-28.8), and Moscow Region – 27.1% (95% CI: 26.0-28.2), significantly higher than in St. Petersburg Region – 22.8% (95% CI: 21.6-24.1).

**Figure 7:**
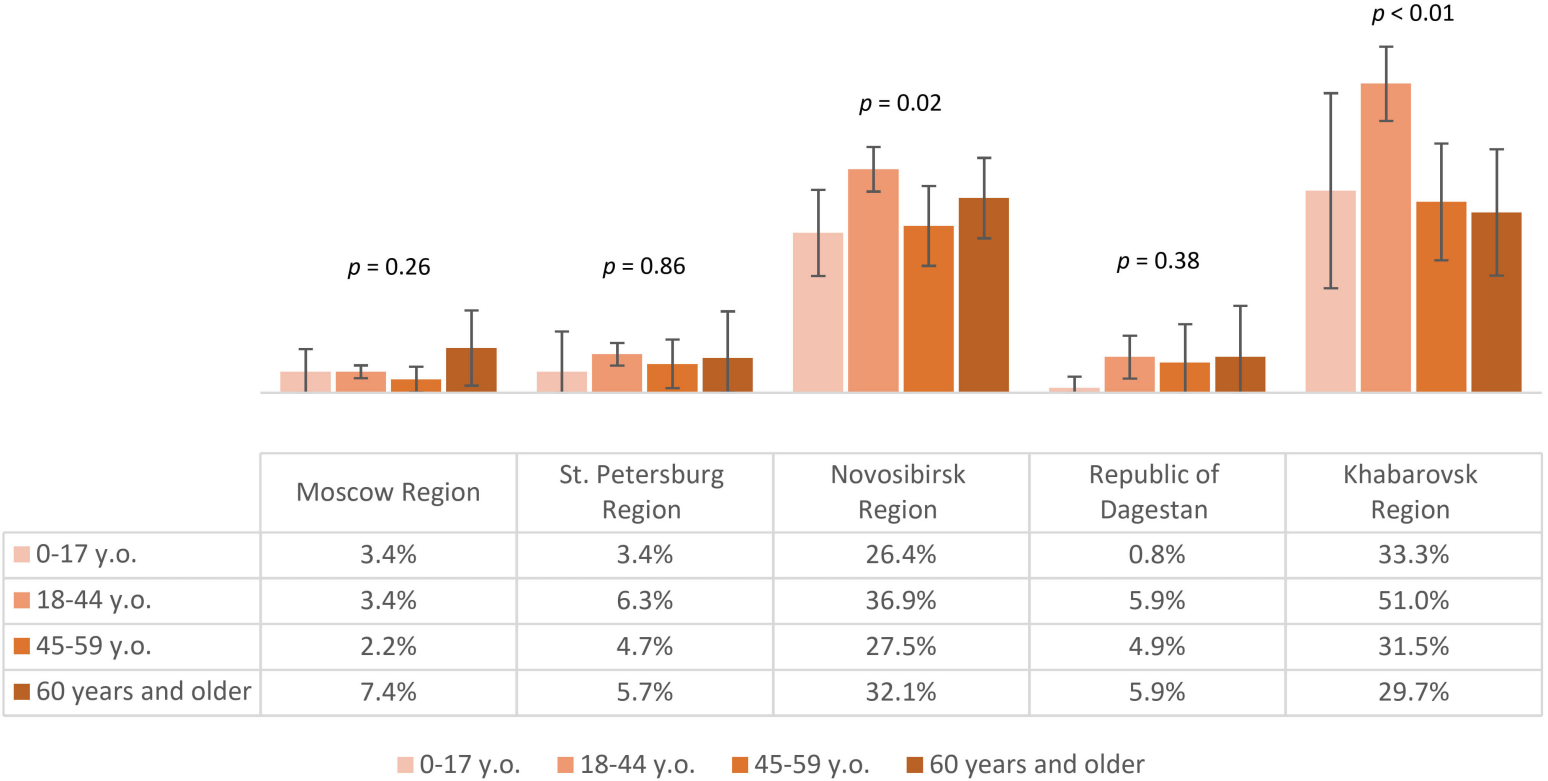
Proportion of non-vaccinated seropositive individuals with tick bites in different regions according to age groups.

Seropositivity analysis of the non-vaccinated population with a history of tick bites demonstrated that the lowest post-infection immunity level was found in Moscow Region – 3.5% (95% CI: 2.5-4.4). The same values were significantly higher in St. Petersburg Region – 6% (95% CI: 4.4-7.6) and the Republic of Dagestan – 6.1% (95% CI: 3.5-8.7). Higher proportions of individuals with post-infection anti-TBEV antibodies were found in Novosibirsk Region – 33.4% (95% CI: 30.7-36.1) and Khabarovsk Region – 42.4% (95% CI: 37.8-46.9), with statistically significant differences between these regions. The seropositivity level of the non-vaccinated population with a history of tick bites in different age groups by region is shown in **Figure 7**. It is important to note that in the regions with the highest observed level of post-infection immunity (Novosibirsk and Khabarovsk Regions), a statistically significant difference was found between age groups, with the relatively higher proportions among individuals aged 18–44 years.

The above results allow us to conclude that in all the selected regions for analysis, the proportion of individuals reporting tick bites was above 15%, with the highest indicators seen in Novosibirsk Region, Khabarovsk Region, and Moscow Region. The highest level of natural post-infection immunity was found in Novosibirsk Region and Khabarovsk Region, especially among those aged 18–44 years.

## Discussion

Our study included various regions of Russia with differing TBE morbidity risk. The epidemiological risk level for each region is calculated based on the average incidence rate per 100,000 population over a 10-year period. In addition to non-endemic Republic of Dagestan, our analysis included regions across all epidemiological risk levels as determined by annual incidence rates from 2014 to 2023. High-risk regions included Novosibirsk Region (annual incidence rate 4.7^о^/_оооо_), medium-risk regions included St. Petersburg (0.89^о^/_оооо_) and Leningrad Region (1.0^о^/_оооо_), and low-risk regions included Khabarovsk Region (0.4^о^/_оооо_) and Moscow Region (0.02^о^/_оооо_) (2).

Since we started collecting blood serum samples from study participants in 2018, TBE cases initially decreased: from 1.03^о^/_оооо_ and 1.02^о^/_оооо_ in 2018–2019 to 0.67^о^/_оооо_ and 0.69^о^/_оооо_ in 2020-2021 (29–32). This was followed by a nearly two-fold increase to 1.34^о^/_оооо_ in 2022 and 1.22^о^/_оооо_ in 2023 (2, 3). Thus, TBE incidence in 2023 was higher than the same indicator in 2014 (2, 3).

Our analysis of seropositivity and protective immunity showed significant geographic differences between the regions studied. The highest level of immunity (seroprevalence with or without protective levels of antibodies) against TBE was found among participants from Novosibirsk Region and Khabarovsk Region. Considering that most of the study participants in these regions represented endemic administrative territories, we concluded that even in relatively better-protected regions, the level of protective immunity was insufficient, especially among children and those aged 60 and older. The situation was even more challenging in St. Petersburg and Leningrad Region where we found low population immunity levels across all age groups.

Similar patterns were observed in other TBE endemic regions: the proportions of seropositive individuals were 61.2% among vaccinated adult donors in the Republic of Altai (a high epidemiological risk region), 23.9% in Chelyabinsk Region, and 13.1% in Zabaykalsky Krai (both medium epidemiological risk regions) (2, 18, 19, 21, 22).

In all studied regions, except the non-endemic Republic of Dagestan, vaccination significantly contributed to the seropositive population. This can be explained by the fact that only Republic of Dagestan is non-endemic among all the regions studied. Therefore, in this region there is lower awareness of TBE as well as there is no mass vaccination campaign for the population. Our data also reveal a notable proportion of individuals with post-infection antibodies across all studied regions.

Our analysis of vaccination coverage showed the highest levels among volunteers from Novosibirsk Region and Khabarovsk Region, correlating with higher seropositivity rates and protective immunity prevalence in these populations. Vaccination rates were particularly low among children and those aged 60 years and older in Novosibirsk and Khabarovsk Regions. Low vaccination rates were also noted across all age groups in St. Petersburg Region.

Most previous studies reported vaccination coverage of less than 50% in endemic regions like Republic of Altai, Chelyabinsk Region, Zabaykalsky Krai, Kemerovo Region, and Kurgan Region (14, 18, 19, 21, 22, 33). The highest vaccination coverage (78%), according to published data, was achieved in Sverdlovsk Region, significantly reducing TBE incidence without pronounced cyclical peaks (13, 34). Our data on the proportion of unvaccinated individuals in the seropositive population and vaccination coverage highlight the inadequacies in planning and implementing the mass vaccination program (1).

Our analysis of post-vaccine immunity showed that the proportion of individuals with protective antibody titers (≥1:400) among vaccinated study participants was far from 100% in most of the regions studied, consistent with results from studies in Zabaykalsky Krai (60.4%) (21, 22). These results may explain the annual cases of TBE observed in the vaccinated population. Despite data on the high immunological efficacy of vaccines used in Russia, the proportion of vaccinated individuals in the TBE incidence structure ranged from 3.7% to 23.8% in various regions (13, 14, 20, 34).

Low TBEV antibody titers in vaccinated individuals may be related to immunization scheme violations or insufficient adherence to revaccination, leading to low protection levels. A study in Kurgan Region indicated that post-vaccine immunity duration and intensity depend on the number of vaccinations and missed revaccinations (14). Conversely, a study in the Republic of Altai reported high seropositivity rates (69.7%) even among those vaccinated over 10 years ago. However, the study authors noted that this was likely due to natural immunization (18). Our study results, excluding individuals with tick bites — a key TBE risk factor — offer a preferable analysis of post-vaccine immunity.

Results of the post-vaccine and post-infection immunity analysis in the studied regions of Russia explain the obtained differences in the level and structure of population immunity. Higher levels of population immunity in Novosibirsk Region and Khabarovsk Region correlate with higher vaccination coverage, and with higher levels of both post-vaccine and post-infection immunity among volunteers from these regions. Across study participants from other regions, differences in population immunity also clearly correlate with vaccination coverage, post-vaccine and post-infection immunity rates.

Our natural post-infection immunity analysis complements existing incidence data, highlighting the importance of regular serological monitoring for effective TBE risk classification (2).

In general, relatively low vaccination coverage and low post-vaccine immunity level according to our study were characteristics of non-endemic region (Republic of Dagestan) and region with low epidemiological risk (Moscow Region). As mentioned above, this may be due to lower public awareness of the risks associated with TBE. However, the results of our study show that low epidemiological risk does not always correlate with lower risk of infection and lower vaccination coverage.

For example, our study of a large sample of volunteers from Khabarovsk Region revealed higher vaccination coverage rates than those in St. Petersburg and Leningrad Region. The average incidence rates per 100,000 were higher in St. Petersburg and Leningrad Region compared to Khabarovsk Region. Meanwhile, rates of virus carriage were lower in St. Petersburg and Leningrad Region (0.2%-0.9%) than in Khabarovsk Region (1.2%) (2, 35–37). Considering the significantly higher proportion of individuals reporting tick bites and significantly higher level of post-infection immunity in Khabarovsk Region than in St. Petersburg and Leningrad Region, the observed differences in population immunity levels, vaccination coverage, and seropositivity rates (including level of protective immunity) may explain the higher epidemic TBE risks yet lower incidence in Khabarovsk Region compared to St. Petersburg and Leningrad Region.

Unexpectedly, the natural post-infection immunity level was higher in Khabarovsk Region compared to Novosibirsk Region, despite lower average incidence, vaccination coverage, and post-vaccine protective immunity level in Khabarovsk Region (2). The virus carriage rate of ticks taken from humans was 1.1-2.3% in Novosibirsk Region and an average of 1.2% in Khabarovsk Region in 2017-2019 (37, 38). This could be due to differences in the pathogenicity of TBEV strains, the diversity of tick populations, the presence of natural foci of other viruses phylogenetically related to TBE, and other reasons.

Similar levels of post-infection immunity in the non-endemic Republic of Dagestan, low-risk Moscow Region, and medium-risk St. Petersburg Region can be attributed to possible attenuation of the existing TBEV foci, especially in the St. Petersburg region, and imported cases of subclinical or inapparent TBE infection. Cross-reactivity with antibodies against West Nile Fever virus, common in Southern Russia, may also play a role (39, 40).

A limitation of our study is the lack of current genetic landscape data and tick virus carriage in the studied regions, as well as the use of ELISA data only. However, it is worth noting that questionnaires, despite certain shortcomings, remain an acceptable method for collecting socio-demographic and anamnestic data.

The study period also covered the COVID-19 pandemic. This reduced people’s contact with natural foci during the epidemiologically dangerous tick activity season. As a result, the number of people affected by tick bites in TBE-endemic regions decreased by 17.7%. Next, healthcare institutions shifted focus to prevent and treat COVID-19. This may have caused underdiagnosis of TBE (29).

Our study provided unique data on the level, structure, and intensity of population immunity against TBEV. We analyzed the characteristics of post-vaccine and post-infection components of population immunity in various age groups across several regions with different epidemiological situations. We highlighted current issues in the effectiveness of the mass vaccination program against TBEV in Russia.

Improving approaches to public education about the importance of vaccine prophylaxis and timely revaccination, enhancing access to TBE vaccination, and developing new vaccines with longer-lasting and broader-spectrum immunological protection can improve population immunity against TBE and reduce the disease burden.

## Conclusions

Our results demonstrated diversity of population immunity level and structure in different regions of Russia. The analysis showed that study participants are at risk of TBE infection, especially high in endemic regions, due to insufficient level of population immunity, vaccination coverage, and protective post-vaccine immunity.

## Data Availability

The raw data supporting the conclusions of this article will be made available by the authors, without undue reservation.

## Supplementary material

Supplementary Table 1 Proportion of study participants of different age groups in certain regions compared to the Russian Federation as a whole.
