# Supplementary material for: Study of the influence of population immunity to tick-borne encephalitis virus on the characteristics of the epidemic process in Russia
Source: Front Immunol. 2025 Aug 8;16:1525388. doi: 10.3389/fimmu.2025.1525388 (PMC12370688; doi:10.3389/fimmu.2025.1525388)
Supplement: Supplementary Table 1 — Proportion of study participants of different age groups in certain regions compared to the Russian Federation as a whole. [file Table1.docx]

# Supplementary data

**Table 1. Proportion of study participants of different age groups in certain regions compared to the Russian Federation as a whole.**

| Age group | Moscow Region | St. Petersburg Region | Novosibirsk Region | Republic of Dagestan | Khabarovsk Region | Russian Federation |
| --- | --- | --- | --- | --- | --- | --- |
| Up to 1 year | 0.00% | 0.00% | 0.21% | 0.32% | 0.08% | 1.00% |
| 1-2 years | 1.33% | 1.33% | 3.30% | 5.26% | 4.48% | 2.21% |
| 3-4 years | 1.67% | 1.91% | 4.19% | 5.56% | 6.42% | 2.58% |
| 5-6 years | 1.70% | 1.46% | 3.80% | 5.63% | 4.82% | 2.59% |
| 7-8 years | 1.70% | 1.48% | 3.61% | 5.31% | 3.53% | 2.53% |
| 9-10 years | 1.12% | 0.92% | 3.64% | 5.18% | 3.27% | 2.35% |
| 11-12 years | 0.90% | 0.92% | 3.56% | 4.68% | 3.61% | 2.25% |
| 13-14 years | 1.03% | 0.62% | 2.89% | 4.26% | 3.72% | 2.03% |
| 15-16 years | 0.86% | 0.69% | 3.36% | 5.11% | 4.69% | 2.04% |
| 17-18 years | 1.06% | 1.02% | 4.35% | 4.26% | 3.64% | 1.95% |
| 19-20 years | 2.30% | 2.23% | 6.00% | 4.43% | 3.95% | 1.96% |
| 21-22 years | 3.96% | 3.73% | 4.02% | 4.41% | 3.33% | 1.85% |
| 23-24 years | 4.97% | 5.12% | 3.78% | 3.16% | 3.38% | 2.06% |
| 25-26 years | 5.82% | 5.87% | 3.09% | 2.86% | 3.06% | 2.10% |
| 27-28 years | 7.07% | 7.37% | 3.51% | 2.77% | 3.64% | 2.54% |
| 29-30 years | 8.34% | 8.68% | 3.88% | 2.72% | 3.53% | 3.25% |
| 31-32 years | 9.28% | 9.70% | 3.98% | 3.09% | 3.82% | 3.45% |
| 33-34 years | 7.96% | 8.57% | 4.30% | 2.99% | 4.06% | 3.55% |
| 35-36 years | 6.56% | 6.60% | 4.25% | 2.37% | 2.93% | 3.39% |
| 37-38 years | 5.92% | 5.20% | 3.38% | 1.84% | 1.75% | 3.14% |
| 39-40 years | 4.52% | 3.98% | 2.71% | 1.37% | 1.73% | 3.18% |
| 41-45 years | 8.00% | 6.87% | 5.01% | 3.24% | 3.74% | 7.22% |
| 46-50 years | 4.58% | 4.81% | 4.56% | 3.01% | 3.33% | 6.53% |
| 51-55 years | 3.40% | 3.66% | 2.90% | 2.74% | 3.77% | 6.11% |
| 56-60 years | 2.78% | 3.33% | 3.26% | 4.16% | 4.29% | 7.29% |
| 61-65 years | 1.74% | 2.00% | 3.31% | 3.24% | 3.74% | 6.81% |
| 66-70 years | 0.97% | 1.27% | 2.78% | 3.36% | 4.43% | 5.50% |
| 70+ years | 0.44% | 0.64% | 2.39% | 2.67% | 3.25% | 8.53% |
